# Reduced Representation Libraries from DNA Pools Analysed with Next Generation Semiconductor Based-Sequencing to Identify SNPs in Extreme and Divergent Pigs for Back Fat Thickness

**DOI:** 10.1155/2015/950737

**Published:** 2015-03-04

**Authors:** Samuele Bovo, Francesca Bertolini, Giuseppina Schiavo, Gianluca Mazzoni, Stefania Dall'Olio, Luca Fontanesi

**Affiliations:** Department of Agricultural and Food Sciences (DISTAL), Division of Animal Sciences, University of Bologna, Viale Fanin 46, 40127 Bologna, Italy

## Abstract

The aim of this study was to identify single nucleotide polymorphisms (SNPs) that could be associated with back fat thickness (BFT) in pigs. To achieve this goal, we evaluated the potential and limits of an experimental design that combined several methodologies. DNA samples from two groups of Italian Large White pigs with divergent estimating breeding value (EBV) for BFT were separately pooled and sequenced, after preparation of reduced representation libraries (RRLs), on the Ion Torrent technology. Taking advantage from SNAPE for SNPs calling in sequenced DNA pools, 39,165 SNPs were identified; 1/4 of them were novel variants not reported in dbSNP. Combining sequencing data with Illumina PorcineSNP60 BeadChip genotyping results on the same animals, 661 genomic positions overlapped with a good approximation of minor allele frequency estimation. A total of 54 SNPs showing enriched alleles in one or in the other RRLs might be potential markers associated with BFT. Some of these SNPs were close to genes involved in obesity related phenotypes.

## 1. Introduction

The pig (*Sus scrofa*) is the most relevant agricultural meat species as well as an important animal model for its numerous physiological and morphological similarities to the human [1]. A parameter that is important for both aspects (meat production and animal model) is the level of fat deposition [2]. This is a complex phenotype that can be evaluated considering different traits. For example, back fat thickness (BFT) is a trait that affects ham and carcass values and, indirectly, correlates with production efficiency. For these reasons, breeding programs in most pig breeds and lines are designed to reduce BFT and increase lean meat content. In a few pig lines, an excessive reduction of the level of BFT could create problems to the meat processing industries as in the case of heavy pigs whose legs are cured for the production of dry-cured hams, and, for this reason, animals are selected to maintain an optimized fat thickness [3]. This trait is also an interesting phenotype to consider the pig as a model for human obesity [4, 5] that is one of the major health problems in both developed and developing countries.

To understand the biological mechanisms affecting BFT in pigs, we recently carried out several studies to elucidate the genetic factors involved in the definition of this trait and to obtain a systems biology comparative picture of human and pig obesity related traits [6]. In a whole genome candidate gene approach, we reported that polymorphisms in genes already shown to affect fat deposition in humans and mice are associated with BFT or correlated traits in commercial pigs and in the Italian Large White heavy pig breed [7–10]. In addition, a genome wide association (GWA) study which we carried out in the same breed using a selective genotyping approach and the Illumina PorcineSNP60 BeadChip [11] showed quite a large number of markers associated with BFT (each with a small effect that could not explain the whole genetic variability for this trait), with a limited overlap with other GWA studies that investigated the same or similar traits in other breeds and pig populations [12]. This could be due to different experimental designs and incomplete power in the different studies as well as different linkage disequilibrium structures of the investigated populations that could not be captured completely by the genotyping tool (Illumina PorcineSNP60 BeadChip).

Taking advantage from the sequenced genome of the pig and its reference assembly (Sscrofa10.2) [13], it is now possible to use next generation sequencing (NGS) platforms to further investigate the level and extent of genetic variability in different breeds and populations (i.e., [14]). The Ion Torrent technology is a cheap promising NGS platform that is based on a semiconductor detection of pH variation during the sequencing process that can be applied in different experimental approaches in which a medium-high throughput is needed [15]. We already evaluated the Ion Torrent platform to analyse a mammalian genome by sequencing reduced representation libraries (RRLs) obtained from rabbit genomic DNA and identified thousands of new single nucleotide polymorphisms (SNPs) in this species [16].

In this study, with the final aim to identify SNPs that could be useful to evaluate the peculiarities of the Italian Large White heavy pig breed and explain, at least in part, the missed genetic variability for the BFT trait not completely captured by our previous association works, we tested the potential and limits of an experimental design in which we combined the Ion Torrent sequencing technology to sequence RRLs. Reduced representation libraries were obtained by enzymatically digest DNA pools constructed from divergent Italian Large White pigs with extreme estimated breeding value (EBV) for BFT. In addition, we used Illumina PorcineSNP60 BeadChip genotyping data already generated from the same animals to obtain a comparative analysis and validation of the sequencing information.

## 2. Materials and Methods

### 2.1. Animals and Genomic DNA

A subset of the Italian Large White pigs that were previously used in a GWA study, carried out to identify markers associated with BFT EBV [12], were used to constitute two genomic DNA pools. The selected animals were from two groups, each of 50 pigs, of two-generation unrelated gilts with extreme and divergent BFT EBV (50 with the most negative BFT EBV and 50 with the most positive BFT EBV), selected among about 12,000 pigs individually performance-tested at the Central Test Station of the National Pig Breeder Association (ANAS) for the sib-testing evaluation of candidate boars within the national selection program of the Italian Large White breed [7, 9, 12]. Average and standard deviation of BFT EBV of the pigs in the negative and positive tails were –9.40 ± 1.60 mm and +8.00 ± 5.95 mm, respectively. Estimated breeding values for this trait were calculated by a BLUP-multiple trait animal model including the fixed factors of batch, age at the beginning of test, date of slaughtering, inbreeding coefficient, body weight at slaughter, and age at slaughter, besides the random factors of animal and litter.

Genomic DNA was extracted from blood using the Wizard Genomic DNA Purification kit (Promega Corporation, Madison, WI, USA). Extracted DNA was quantified using a NanoPhotometer P-330 instrument (Implen GmbH, München, Germany) and pooled at equimolar concentration to constitute two DNA pools, one including DNA from the 50 Italian Large White pigs with the lowest BFT EBV and a second including DNA from the 50 Italian Large White pigs with the highest BFT EBV.

### 2.2. Genotyping

The investigated animals were previously genotyped with the Illumina PorcineSNP60 BeadChip (Illumina Inc., San Diego, CA, USA), interrogating 62,163 SNPs [11]. No filter was applied and all samples and genomic positions were retained for subsequent evaluation and comparison with sequencing data (see below).

### 2.3. Reduced Representation Libraries

Ten micrograms of DNA from each of the two pools were digested overnight with 50 U of* Hae*III restriction enzyme and the digested products were loaded in a 0.8% agarose gel.* Hae*III was selected as it did not produce visible patterns that could be ascribed to repetitive elements in the range of 500–700 bp (data not shown). DNA fragments from this range obtained from* Hae*III digestion were purified from the agarose gel with the QIAquick Gel Extraction Kit (Qiagen, Hilden, Germany) according to the manufacturer instructions. Obtained DNA was used for library preparation and sequencing with the Ion Torrent PGM (Life Technologies, Carlsbad, CA, USA).

### 2.4. Ion Torrent Sequencing

Sequencing of the two RRLs was obtained using 200 ng of DNA that was purified by agarose gel electrophoresis as described above, enzymatically sheared, end-repaired, and adapter-ligated using the Ion Xpress Plus Fragment Library Kit (Life Technologies). Obtained DNA material was size-selected using the e-gel system (Invitrogen, Carlsbad, CA, USA) and bands corresponding to 100 bp of inserts were collected and quantified by qPCR using a StepOnePlus Real-Time PCR System (Life Technologies). Selected fragments were clonally amplified, purified, and sequenced using the Ion One Touch 100 Template Kit and the Ion PGM Sequencing Kit with two Ion 318 chips (Life Technologies), for the two RRLs.

### 2.5. Sequence Data Analyses

Obtained sequencing reads were filtered and trimmed using the Ion Torrent suite v.2.2 (Life Technologies) which (i) eliminated polyclonal sequences and sequences of low quality and (ii) trimmed adapters and low quality 3′-ends. Then data were inspected with FastQC v.0.11.22 [17]. Sequenced reads were trimmed and filtered using PRINSEQ Lite v.0.20.4 [18] as follows: (i) trimming at the 3′-end up to 140 bp, (ii) trimming of the 5′-end and 3′-end for poly-A/T sequences > 5, (iii) trimming the 5′-end and 3′-end up to reaching a base with a quality score > 20, (iv) exclusion of reads having average quality < 20, and (v) exclusion of reads shorter than 20 bp. PCR duplicates were removed from each library using Picard v. 1.107 [19]. After the PCR duplicates removing step, reads were merged, processed, and aligned on the Sscrofa10.2 genome version using BWA v.0.7.7 [18]. Reads aligning in only one place of the genome and with mapping quality score (Qm) > 20 were retained. SNP calling was obtained using SNAPE [20], setting divergence to 0.01, prior nucleotide diversity (*θ*) of 0.001, folded spectrum, and filtering by a posterior probability of segregation > 0.90. SNAPE input files (PILEUP format) were obtained using Samtools v.0.1.4 [21, 22]. SNAPE filters were applied to consider only positions with minimum depth of 3x, to avoid indels (as indel calling algorithm is not specific for pools [23]). For each putative SNP, we identified if it was already included in the dbSNP or if it was new using the Ensembl BioMart data mining tool [24], interrogating the Ensembl Variation 77 database (October 2014) for Sscrofa10.2 short variations and indels (based on dbSNP build 140). All the SNPs that did not match with those reported on dbSNPs were also analyzed with the Samtools mpileup function [21, 22]. Variant effect predictor (VEP) tool (http://www.ensembl.org/Sus_scrofa/Tools/VEP; [25]) was used to map gene positions and to predict the effect of each substitution. SIFT [26] was used to evaluate if missense mutations could have deleterious effects on the translated proteins.

In order to evaluate differences in allele frequency derived by the number of alternative reads between the two RRLs, Fisher's exact test was computed for each alternative genomic position covered by a minimum depth of 3x. All the positions with *P*_Fisher_ < 0.05 were also visually inspected with IGV (Integrative Genomics Viewer) software [27].

## 3. Results

### 3.1. Sequencing Data and Identification of SNPs

A total of 3,390,796 and 3,731,776 sequenced reads were obtained from the two RRLs produced using the positive and negative BFT EBV DNA pools, respectively (Table 1). After cleaning the datasets for duplicated reads, the number of unique reads was 2,692,605 and 2,885,815, respectively (Table 1). A total of 1,449,838 (positive BFT EBV RRL) and 1,476,125 (negative BFT EBV RRL) reads were mapped with high confidence to the Sscrofa10.2 assembly of the pig genome. The merged dataset had an average read depth (RD) of 1.28x (range from 1 to 426x). Table S1 (see Supplementary Material available online at http://dx.doi.org/10.1155/2015/950737) reports the number of reads and nucleotides mapped on the different pig chromosomes. Sequence data obtained from the two RRLs have been submitted to the European Nucleotide Archive database (EMBL, http://www.ebi.ac.uk/ena/) and are indexed with the accession number ERP009239.

Using sequencing data, a total of 39,165 putative SNPs were called with high confidence by SNAPE [20]. Of these SNPs 24,560 (62.5%) were polymorphic carrying two alleles within the sequenced reads and 14,605 (37.58%) were monomorphic for an alternative form than that of the reference genome. We detected 9,680 new putative SNPs not yet reported in dbSNP (24.72% of the called SNPs) while the major part of identified variations (29,485; 75.28%) was already present in dbSNP. The transition/transversion ratio considering all the detected SNPs is 2.08, comparable to other mammalian genomes [28]. In addition, 6,324 of the newly detected SNPs were also detected using Samtools and 3,964 of these SNPs had score ≥20.  Table 2 reports the summary of the annotations of the identified SNPs. Most of the SNPs were in intergenic (56.1%) or in intronic (28.9%) regions. The list of SNPs included in transcribed regions is reported in Table S2. Among the putative SNPs predicted in coding regions, 217 were synonymous mutations, 159 were missense mutations, two were stop-gained mutations (in the novel gene ENSSSCG00000028324 and in the NUT family member 2D gene, known as* NUTM2D*), and one was a stop-lost variation (in the putative pleckstrin and Sec7 domain containing 2 gene;* PS2D*). Among the missense mutations, 37 were considered deleterious by SIFT (Table S2). Several genes with deleterious missense mutations (e.g., NADH dehydrogenase (ubiquinone) 1, subcomplex unknown, 1, 6kDa (*NDUFC1*); parathyroid hormone 1 receptor (*PTH1R*); glycerol-3-phosphate acyltransferase 2, mitochondrial (*GPAT2*); and several olfactory receptor like genes) play important roles in different biochemical and physiological cellular mechanisms.

### 3.2. Sequencing versus PorcineSNP60 BeadChip Genotyping Data

To validate some of the called SNPs we took advantage from the Illumina PorcineSNP60 BeadChip genotyping data obtained on the same animals used to construct the two RRLs. Considering SNP positions covered by a minimum of three reads, 661 out of 62,163 SNPs of the chip (1.1%) were identified from the 13,596,939 sequenced positions (0.45% of the porcine genome). SNAPE analysis over these positions reported that (i) 3 positions were discarded and 8 had read depth < 3 (for further features of SNAPE in addition to the general criteria adopted), (ii) 257 were identified as SNPs (152 polymorphic SNPs carrying two alleles while 105 SNPs were monomorphic for an alternative form from that of the reference genome), and (iii) 375 positions showed only the sequence of the reference genome.

Of the overlapping 653 positions (661 – 8 = 653), (i) for 28 of them the chip genotype data of the individual pigs were not possible to retrieve (probably due to problems in the design of the chip probes that could prevent the genotyping) and (ii) for 63 DNA positions having all individuals homozygous for only one genotype 59 of these base positions matched with the genotype inferred by NGS, whereas 2 were called as heterozygous and 2 were called as homozygous for a noncomplementary nucleotide by sequencing data (Table S3). If we go into more details for the 28 SNPs that failed to report reliable genotyping data from the PorcineSNP60 BeadChip, for 12 out of 28 both alleles were present in the NGS reads; 15 out of 28 showed only one allele and one was an erroneous SNP.

**Figure 1:**
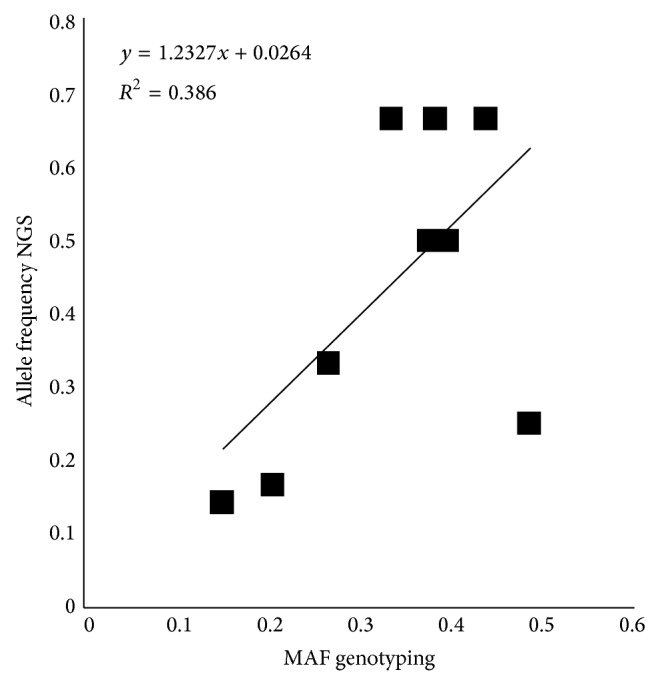
Scatter plot of allele frequency estimated by Ion Torrent sequencing data for SNPs called by at least 6 reads (allele frequency NGS) and obtained by genotyping data (MAF genotyping) for the same SNPs.

In addition to these overlaps between NGS sequencing and genotyping data, we wanted to evaluate if the estimated allele frequencies derived by NGS in RRLs obtained from DNA pools could match the true allele frequencies at the same positions obtained by using the PorcineSNP60 BeadChip. Starting from 559 SNPs (derived by the subsequent filtering steps of the 661 SNPs reported above), 262 (145 called SNPs by SNAPE) had the same type of substitution. Excluding the transversions GC *↔* CG and AT *↔* TA, for each one of the remaining 258 SNPs, we compared the minor allele frequency (MAF) of the genotyping data against the frequency of the same allele derived by the sequencing. Results of the regression analysis are reported in Table 3 and in Figure 1. As expected, a low correlation from these two data was observed when considering all 258 SNPs due to the low coverage depth (3x) that was not enough for a reliable allele frequency estimation from NGS data. This value increased up to 3 times setting a coverage depth equal to or higher than the double of the minimum coverage depth (≥6). When adding data coming from monomorphic allele, correlation increased up to 0.70. These data indicate that even using a coverage depth ≥6 the MAF of these SNPs can be estimated with good approximation.

### 3.3. Sequencing Derived SNPs: Differences between the Two Libraries

For each of the two initial pileups we filtered out genomic positions having depth < 3x and then we used SNAPE to extract the allele frequency of each genomic position taking the advantage of the filters implemented in it. Polymorphic positions were compared among the 237,969 positions that were in common between the two RRLs (Table 1). Among these nucleotides, 67 genomic positions (filtered to 54 when tested by SNAPE and inspected with IGV) showed a *P*_Fisher_ < 0.05 comparing alternative reads observed in the two RRLs generated from DNA of pigs with extreme and divergent BFT EBV (Table S4). Only one of these SNPs showed a *P*_Fisher_ < 0.01. However, no one remained significant after Bonferroni correction. These SNPs were located in several autosomal chromosomes (SSC1, SSC3, SSC6, SSC8, SSC9, SSC10, SSC12, SSC15, SSC16, SSC17, and SSC18). These variants (only 12 of which already deposited in dbSNP) were localized as follows: 63% were intergenic variants, 21% were in introns, 11% were upstream gene variants, and 5% were downstream gene variants. Intronic variants were located in four genes of which only two were annotated with a known function: (1) dysbindin (dystrobrevin binding protein 1) domain containing 1 (*DBNDD1*); (2) phosphatidic acid phosphatase type 2A (*PPAP2A*).

### 3.4. Comparison with Genome Wide Association Results

In order to evaluate if the 54 SNPs that showed differences in number of alternative reads between the two RRLs were located in chromosome regions associated with BFT in Italian Large White pigs (listed in Table S4), we compared their positions on the basis of our previous GWA study carried out in the same breed [12]. We considered a window spanning ± 0.5 Mbp from each marker having nominal *P* value < 0.05 in our previous study [12]. The top *P*_Fisher_ for each of the identified regions is reported in Table 4 (the complete list is reported in Table S5). The most significant marker (M1GA0008302; *P* = 1.65*E* − 06) is located 72,572 bp downstream SNP SSC6:859837 (*P*_Fisher_ = 0.038) and 85,796 bp downstream the 6th top SNP SSC6:873061 (*P*_Fisher_ = 0.012) obtained from the list of the 54 SNPs. In this region there is the acyl-CoA synthetase family member 3 (*ACSF3*) gene that belongs to a family of enzymes that activate fatty acids. In the same region we previously showed that other markers (M1GA0008329, SSC6:996248, *P* = 9.35*E* − 05, and M1GA0008318, SSC6:945991, *P* = 4.41*E* − 04) were associated with BFT in the same breed. Within Table 4, the second most significant marker as reported previously (ALGA0000014, *P* = 1.74*E* − 05 [10]) is located close to the SNP SSC1:68514 (*P*_Fisher_ = 0.029) identified in the present study (Table 4). In this region there is another marker associated with BFT in the previous GWA study (ALGA0000009, *P* = 2.75*E* − 03; [12]). An interesting gene located in this part of the pig genome [12], delta-like 1 (*Drosophila*) (*DLL1*), seems associated to type 1 diabetes in humans. For marker DRGA009307 (SSC9:17138159, *P* = 8.66*E* − 04) there is no annotated gene in a ± 500 kbp region. DIAS0000309 (SSC12:48865200, *P* = 9.96*E* − 04) is near the active breakpoint cluster-related (*ABR*) gene and ENSSSCG00000017808 gene, orthologous of the acyl-CoA-binding protein (*DBI*) gene.* ABR* gene is annotated with two interesting gene ontology (GO) terms: phospholipid binding and brain development.* DBI* gene functions as intracellular carrier of acyl-CoA esters and it seems that it could act as a neuropeptide modulating the action of the GABA receptor. It is annotated with the GO terms: long-chain fatty acyl-CoA binding, transport, phosphatidylcholine acyl-chain remodeling, and triglyceride metabolic process that might suggest a potential role in fat metabolism and deposition.

## 4. Discussion

Next generation sequencing is changing the way to identify markers associated with production traits in livestock species. Several applications and strategies have been designed mainly using Illumina platforms (i.e., [14]). To our knowledge, this study applied for the first time the Ion Torrent technology to identify DNA polymorphisms in the pig genome. The experimental design was quite simple as, at this stage, we wanted to test this NGS technology to identify markers that could be useful for subsequent association studies in the Italian Large White pig breed. The identification of polymorphisms was based on the construction and sequencing of two RRLs generated from DNA pools of pigs with extreme and divergent BFT EBV. This approach was tested to set up a strategy for the identification of polymorphisms at a reduced fraction of the cost required for individual sequencing. In this way, we could also identify variants that might be enriched in one pool compared to the other one. To call SNPs we used SNAPE that is a software package that implemented a Bayesian approach for SNP identification and MAF estimation in sequenced pools [20]. The validation of identified SNPs was obtained by comparing the genotyping data generated with the Illumina PorcineSNP60 BeadChip on the same animals. As we sequenced DNA in pools and genotyping data were obtained on individual animals, we evaluated how allele frequency correlated between the two approaches varying the depth of sequencing. This approach was able to define an interesting procedure to validate SNPs identified from DNA pools.

Reduced representation libraries were generated as a simple strategy to reduce the complexity of mammalian genomes and to obtain information from a small part of it that can be sampled after restriction fragment digestion [29]. Several studies have already applied this strategy in farm animals for SNP discovery [16, 30–32]. For example, in pigs, Wiedmann et al. [31] and Ramos et al. [11] sequenced RRLs for the identification of SNPs that were used to construct the Illumina PorcineSNP60 BeadChip genotyping platform. In our study, we identified about 40k SNPs in the pig genome. This is a quite large number of SNPs, considering the limited throughput of the benchtop Ion Torrent technology (compared to Illumina platforms [33]) and the stringent criteria that we used to call SNPs. As the technology is prone to errors in case of homopolymeric regions [34], indels were not considered in this study. That means that we could probably have discovered other short variants but we did not consider them to guarantee a high quality of the called polymorphisms. In addition, other bioinformatics tools should be developed to obtain a reliable MAF estimation of indels from sequencing data generated from DNA pools [22].

Among the 159 SNPs causing missense mutations, 37 were predicted to affect the function of the encoded protein (Table S2). These polymorphisms will be prioritized to evaluate their association with several production traits together with SNPs whose alleles were differentially enriched in the two RRLs (Table 4, Table S4, and Table S5). The identification of these latter SNPs was based on allele frequency generated by mapping alternative reads in the two extreme groups of pigs with divergent BFT EBV. The low coverage of many SNP positions in both RRLs limited the possibility to identify markers associated with this trait. This problem is also due to the incomplete overlapping of read coverage between the two RRLs. However, a comparative analysis of the nominally significant SNPs with our previous GWA study for BFT obtained using the same animals analyzed in this study [12] indirectly supported, to some extent, the identified association results. Some of these markers were located close to genes already shown in humans and mouse to be involved in obesity related phenotypes and pathologies suggesting a potential effect of these polymorphisms on BFT and fat deposition in Italian Large White pigs. These indications should be supported by association studies with fat deposition traits in the investigated breed or in other pig populations.

## 5. Conclusion

Several methodological approaches were tested in this study for the first time: (i) partial sequencing obtained with Ion Torrent technology of the pig genome from DNA pools by using RRLs; (ii) the application of SNP calling and MAF estimation on Ion Torrent low coverage sequencing data from DNA pools; (iii) the validation of SNP called in DNA pools using individual genotyping data from the same animals of the pools; (iv) the possibility to identify enriched alleles in the two sequenced RRLs representing two extremes for important phenotypes (BFT). All these approaches were implemented in a case study that tried to identify additional markers associated with BFT in the Italian Large White pig breed. The purpose was to set up a strategy that could reduce as much as possible the sequencing cost and that could produce data useful to identify novel markers for the targeted trait. Association studies will be carried out to evaluate the effects of the 54 selected markers.

Ion Torrent can be successfully applied for SNP discovery even if its limited throughput reduced the possibilities to obtain reliable allele frequencies in the two DNA pools. Other reductionist approaches, like genotyping by sequencing or genotyping by genome reducing and sequencing [35, 36], might be used to identify and validate SNPs associated with BFT.

## Supplementary Material

Table S1: Number of sequenced reads and nucleotides mapped on the different pig chromosomes.
Table S2: List of SNPs included in transcribed regions.
Table S3: Overlapping positions between sequencing and PorcineSNP60 Beadchip genotyping data.
Table S4: List of the 54 SNPs showing significant differences (P<0.05) between the two libraries.
Table S5: List of SNPs that showed differences in number of alternative reads compared with the genome wide association results.

## Figures and Tables

**Table 1:** Summary of sequencing data obtained from the two reduced representation libraries (RRLs) of the positive (Pos_*Hae*III) and negative (Neg_*Hae*III) back fat thickness estimated breeding value DNA pools.

Information^1^	Pos_*Hae*III	Neg_*Hae*III	Pos + Neg *Hae*III
Sequenced reads	3,581,496	3,887,066	7,468,562
Reads after preprocessing	3,390,796	3,731,776	7,122,572
Removed duplicates	698,191	845,961	1,544,152
Mapped reads (Qm > 20; Rdup)	1,449,838	1,476,125	2,925,963
Sequenced bases (Qm > 20; Rdup)	137,429,598	145,859,611	256,880,473
Mean and max depth of coverage (Qm > 20; Rdup)	1.18; 209	1.16; 217	1.29; 426
Sequenced bases (Qm > 20; RD ≥ 3; Rdup)	3,394,898	3,057,171	3,942,266
Sequenced bases retained by SNAPE (Qm > 20; RD ≥ 3; Rdup)	3,369,555	3,034,731	237,969 (in common)
SNPs (Qm > 20; RD ≥ 3; Rdup)	10,694	10,339	39,165

^1^Qm = mapping quality; RD = read depth; Rdup = removed duplicates.

**Table 2:** Summary of the SNP annotation results obtained using the variant effect predictor (VEP) tool.

Gene position or SNP effect	Number of SNPs
3 prime UTR variant	203
3 prime UTR variant, NMD transcript variant	1
5 prime UTR variant	58
Downstream gene variant	2710
Intergenic variant	24414
Intron variant	12591
Intron variant, NMD transcript variant	126
Intron variant, noncoding transcript variant	306
Missense variant	159
Missense variant, splice region variant	8
Noncoding transcript exon variant, noncoding transcript variant	29
Splice acceptor variant	2
Splice donor variant	1
Splice region variant, 3 prime UTR variant	1
Splice region variant, intron variant	25
Splice region variant, synonymous variant	12
Stop gained	2
Stop lost	1
Stop retained variant	1
Synonymous variant	217
Synonymous variant, NMD transcript variant	3
Upstream gene variant	2675
Total^*^	43545

^*^The sum includes 39,165 variations, 4,380 of which have multiple annotations, for a total of 43,545 SNP annotations.

**Table 3:** Summary of regression analysis between allele frequency estimated by Ion Torrent sequencing and the allele frequency obtained by genotyping with the Illumina PorcineSNP60 BeadChip.

RD	Polymorphic sites		Polymorphic and monomorphic sites	
	*R* ^2^	Positions	*R* ^2^	Positions
≥3	0.1199	258	0.6882	317 (258 + 59)
≥4	0.1601	99	0.6399	119 (99 + 20)
≥5	0.1611	36	0.5868	41 (36 + 5)
≥6	0.3866	11	0.7006	13 (11 + 2)

RD = read depth; *R* ^2^ = regression coefficient; positions: number of genomic sites analyzed.

**Table 4:** Overlapping results between the SNPs associated with back fat thickness as identified with the Ion Torrent sequencing data (*P*_Fisher_ < 0.05) and those obtained in the genome wide association study (GWAS) reported by Fontanesi et al. [10] (*P* < 0.05, window = ±0.5 Mbp for each marker).

Chr.	Marker	Pos_M_	P_GWAS_	Pos_SNP_	*P*_Fisher_^*^
1	ALGA0000009	52,297	2.75*E* − 03	68,514	2.86*E* − 02
1	ALGA0000014	79,763	1.74*E* − 05	68,514	2.86*E* − 02
6	M1GA0008302	787,265	1.65*E* − 06	873,061	1.28*E* − 02
6	M1GA0008318	945,991	4.41*E* − 04	873,061	1.28*E* − 02
6	M1GA0008329	996,248	9.35*E* − 05	873,061	1.28*E* − 02
9	DRGA0009307	17,138,159	8.66*E* − 04	16,885,924	2.81*E* − 02
12	DIAS0000309	48,865,200	9.96*E* − 04	48,937,212	2.63*E* − 02

^*^Only the top *P*_Fisher_ for each marker is listed. All other data are presented in Table S5.

Chr. = chromosome; marker = marker in the Illumina PorcineSNP60 BeadChip; Pos_M_ = nucleotide position of the marker on the Sscrofa10.2 reference genome; *P*_GWAS_ = *P* value of association in the GWAS; Pos_SNP_ = nucleotide position on the Sscrofa10.2 reference genome of the SNP having *P*_Fisher_ < 0.05; *P*_Fisher_ = *P* value of Fisher's test.
